# Ultrafast photoinduced dynamics of a donor-($\mathrm \pi$)bridge-acceptor based merocyanine dye

**DOI:** 10.1038/s41598-022-23080-5

**Published:** 2022-10-29

**Authors:** Jianwei Shen, Ajay Jha, Meng Lv, Guanyu Jiang, Qixu Zhao, Zihui Liu, Jinquan Chen, Yan Yang, Haitao Sun, Hong-Guang Duan, Zhenrong Sun

**Affiliations:** 1 State Key Laboratory of Precision Spectroscopy, School of Physics and Electronic Science, East China Normal University, Shanghai, 200241 People’s Republic of China; 2 The Rosalind Franklin Institute, Rutherford Appleton Laboratory, Harwell Campus, Didcot, Oxfordshire, OX11 OFA UK; 3 Department of Physics and Institute of Modern Physics, Ningbo University, Ningbo, 315211 China

**Keywords:** Physical chemistry, Solar cells

## Abstract

Merocyanine dyes are of great interest amongst researchers due to their nonlinear optical (NLO) properties and solvatochromism. Molecular structure of these dyes constitutes conjugated pathway between the donor and acceptor substituents, with lowest energy transition of $\mathrm \pi$–$\mathrm \pi$* character. To rationalize the design of these dyes and deduce structure-property relationship, it is eminent to unravel the excited state dynamics in these complex molecular structures in different solvents. Here we have studied excited state dynamics of a merocyanine dye known as HB194, which has shown commendable efficiency in small molecule based bulk heterojuction solar cells. We have employed femtosecond transient absorption in combination with the quantum chemistry calculations to unravel the solvent dependent charge transfer dynamics of HB194. The excited state decays of the HB194 in different solvents show multi-exponential components. The analysis of the time-resolved data reveals that the polar solvents induce conformationally relaxed intramolecular charge transfer state. In non-polar solvent cyclohexane, only solvent-stabilized ICT state is observed. Additionally, we observe an anomalously red-shifted emission in ethylene glycol centred at $\sim$ 750 nm. Our computational calculations suggest the presence of molecular dimers resulting into observed red-shifted emission band. Our work therefore underscores the importance of gathering molecular-level insight into the system-bath interactions for designing next generation merocynanine-based solvatochromic dyes.

Covalently connected donor-acceptor molecules are designed and tuned to attain charge-transfer-mediated structural rearrangements, which manifests solvatochromic effects to be used for various applications^1–4^. Merocyanine dyes with donor-($\pi$)bridge-acceptor backbone belong to the class of neutral polymethine dyes, where one end is analogous to cyanine dyes and the second is taken from an active methylene compound^5,6^. The specific electron accepting/donating capabilities of the two ends of these dyes along with the bridge dictate the electronic structure and solvatochromic effects, which make these dyes useful for environment sensing in live cell imaging applications^7^. On account of their high dipole moment, absorption coefficients and polarizabilities, merocyanine dyes are also used for photorefractive materials and nonlinear optics applications^8–11^. To rationally improve the molecular design for various applications it is important to understand the underlying photophysics of the prevalent merocyanine dyes.

In recent years, merocyanine dyes have also been shown as promising substitutes for ruthenium complex based dyes in Graetzel-type dye-sensitized solar cells. This has further gained attention towards understanding the photophysics of these merocyanine dyes^12–19^. Computational studies on merocyanines containing $\alpha$-cyano carboxylic acid groups as electron acceptors and triarylamine groups as donors have shown that the lowest energy transitions, $S_0$ $\rightarrow$ $S_1$ is dominantly described as $\mathrm \pi$–$\mathrm \pi$*, with rather weak CT character^20^. As the length of the bridge increases, the CT character of this transitions increases. The role of torsional coordinate after primary photoexcitation has also been predicted^21–23^. Considering the partial CT character of the lowest energy transition, it is expected for these dyes to shown solvatochromic behaviour. Additionally, molecular aggregation of merocynanine dyes in solution has also been reported using various spectroscopic studies^10,24,25^. Considering the important role of solvent in determining molecular properties of these dyes, it becomes essential to understand the photoinduced dynamics of these dyes in different solvent conditions. Here, we have studied one of the merocyanine dyes HB194; 2-((Z)-2-((E)-2-(1,1-Dimethyl-5,6-dihydro-1H-pyrrolo[3,2,1-ij] quinolin-2(4H)-ylidene)ethylidene)-3-oxo-2,3-dihydro-1H-inden-1-ylidene) malononitrile, which has been employed for solar cell application^26^. A simple layer stack small molecule based bulk heterojuction solar cell based on the HB194 delivered a promising photon conversion efficiency of 6.1%. This efficiency seems very low as compared to the current state-of-the-art solar cells containing dyes such as dye-sensitized solar cells, but in case of stacked small molecule based bulk heterojunction cells 6.1% efficiency is noteworthy. The measurement of solar cell power conversion efficiencies does not capture the molecular basis for success or failure of the dye. This further motivates us to understand the underlying photophysics of HB194 molecular system, which can direct rational modification of the dye to obtain higher efficiency in bulk heterojuction solar cells. Here, we have employed steady state absorption and emission measurements along with the computational calculations to highlight the solvatochromic behaviour in HB194. To unravel the interplay of different excited states and their lifetimes of HB194 dye in various solvents, we have employed ultrafast transient absorption measurements in visible region of the spectrum (450–750 nm). Detailed analysis of the time-resolved data reveals the role of the solvents in determining the lifetime of the underlying excited states. Also, HB194 shows uniquely red-shifted emission in ethylene glycol, which is assigned to the presence of molecular dimers in the solution using computational calculations. Excited state dynamics of the molecular dimer in ethylene glycol has also been described.

## Results

The solution of HB194 with different solvents are prepared in a cuvette cell with optical pathlength of 1 mm. The steady-state absorption and fluorescence spectra are measured on a commercial optical setup. For the transient absorption measurements, the cuvette is mounted in a XYZ translation stage to adjust the position on the focusing point between pump and probe beams. Detailed description of HB194 solution is presented in the “Materials and methods” section.

### Steady-state absorption and emission measurements

**Figure 1:**
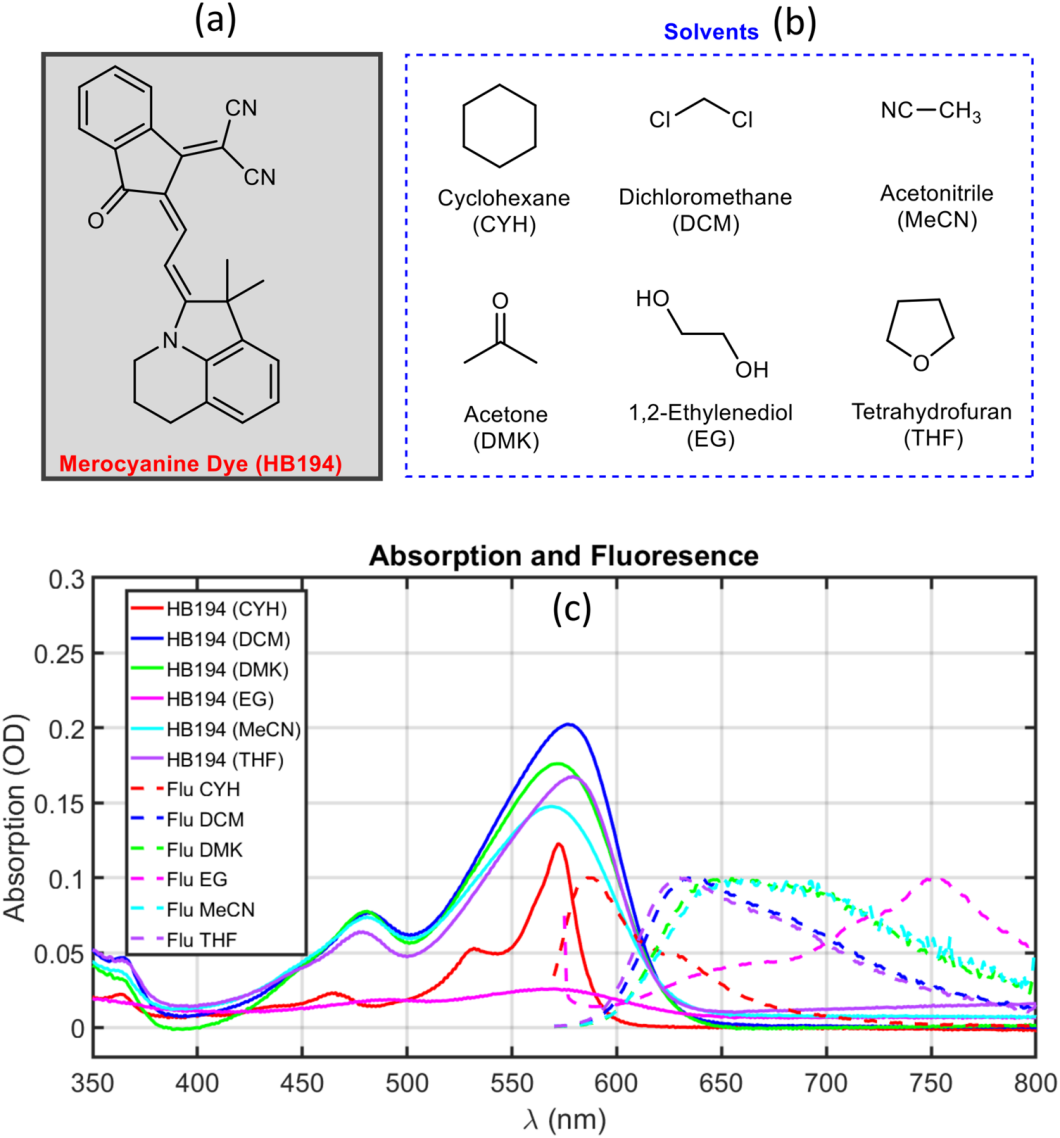
Solvatochromism in Merocyanine dye, HB194. (**a**) Molecular structure of Merocyanine dye (HB194); (**b**) solvents used in this study to understand the solvatochromic behaviour of the dye; (**c**) absorption (solid lines) and fluorescence spectra (dashed lines) of HB194 in different solvents. The absorption spectra of HB194 solutions are shown in optical density (OD) unit, which are used for transient absorption studies. The fluorescence spectra are obtained after subjecting the sample solutions to excitation at 530 nm and then the spectra are normalized for comparison. The solvatochromic behaviour can clearly be seen from fluorescence spectra with an anomalously red-shifted spectrum in EG. Based on these steady state measurements, HB194 solutions are categorized into four different groups: HB194 (CYH), HB194 (THF, DCM), HB194 (DMK, MeCN) and HB194 (EG).

The steady-state absorption spectra are measured in a commercial spectrometer (TU1901, Beijing Purkinje General Instrument Co. Ltd, UV-Vis spectrophotometer). The measured absorption data of HB194 in different solvents are presented in Fig. 1 as solid lines. The absorption spectrum of HB194 in cyclohexane shows distinct sharp spectral features as compared to the rest of the solvents, possibly hints towards the importance of system-bath interactions in molecule’s photophysics. This obervation is further explored in the computational section. On the basis of absorption measurements, we clearly identify the prominent electronic transitions of HB194 in the visible range of the spectrum in different solvents. The most intense transition is located at 570 nm, which should manifests the lowest energy electronic transition from ground to the low lying excited state. The lowest energy transition in HB194 dye is conjectured to have partial charge transfer (CT) character. The nature of the lowest energy transition has been later explained in the paper using quantum chemistry calculations. An additional side peak is observed at 530 nm (extremely prominent in red solid line, HB194 (CYH)), which indicates a strong vibronic coupling of a particular vibrational mode (around 1390 cm$^{-1}$, possibly represents aromatic ring stretching vibrations or the vibrational mode of cyclohexane) to the electronic transition ($\mathrm S_{0} \rightarrow S_{1}$) and cause the observed spectral profile of vibrational progression. Additionally, the absorption peak at 480 nm manifests the electronic transition from the ground to the second excited state ($\mathrm S_{0}\rightarrow S_{2}$). The nature of this transition has also been further explained in the computational section.

The electronic transition (deactivation) from the partial charge transfer (CT) state to the electronically ground state can be directly resolved by fluorescence spectra. The fluorescence spectra in different solvents are presented in Fig. 1 as dashed lines and the magnitude of the spectra are normalized to 0.1. In Fig. 1, the most intense fluorescence feature at 580 nm (red dashed line, HB194 (CYH)) shows the transition from the conjectured partial CT state to ground state. Furthermore, the dashed purple and blue lines (HB194 with (THF and DCM)) show the main peak at 630 nm with a smaller side band at 680 nm. The emission spectra (dashed green and light blue lines) of HB194 (DMK) and (MeCN) is slightly red-shifted to 650 nm with a small side band at 700 nm. It should be noted that we do observe the emission from the excited state, the relatively strong emission bands infer the nature of lowest energy transition is only partially charge transfer in character. However, the exceptional case of HB194 (EG) (dashed purple line) presents a dramatic bathochromic shift of the emission band. The maximum magnitude of the emission band in EG is located at 750 nm.

On the basis of the steady-state absorption and emission spectra, we can clearly point out that the absorption peaks are very similar while the emission peaks are significantly solvent-dependent. Using these measurements, we can categorize these steady-state optical data into four different cases on the basis of underlying reorganization energies after CT excitation. The reorganization energy usually corresponds to half the energy difference between absorption and emission peaks. The first case, HB194 (CYH) shows absorption spectrum with the most narrow bands, rendering the clear observation of vibrational progression. Additionally, the fluorescence spectrum (dashed red line) also show two resolvable peaks at 580 and 630 nm, respectively. The HB194 (DCM) and (THF) are sorted into the second case. The observed bandwidth of the peaks in this category is relatively larger compare to first case of HB194 (CYH), which is reasonable considering the stronger interaction between HB194 and solvent molecules in second case (DCM, dielectric constant, 8.92). In addition, the main peak of fluorescence spectra are red-shifted to 630 nm and the side peaks locate at 680 nm. The HB194 (DMK) and (MeCN) are sorted into the third case, in which, the main transitions of fluorescence spectra are shifted to 650 nm with a side band at 700 nm (DMK, dielectric constant 20.7). The last case is the absorption and fluorescence spectra of HB194 (EG), where the main peak of fluorescence spectrum is dramatically shifted to 750 nm due to a strong interaction to the polar environment (EG, dielectric constant 37.0). To minimize the effort with time-resolvent measurement and data analysis of redundant solvent systems, we have selected the representative solvents to investigate the transient dynamics of electronically excited states and understand the role of CT states.

### Transient absorption spectra

**Figure 2:**
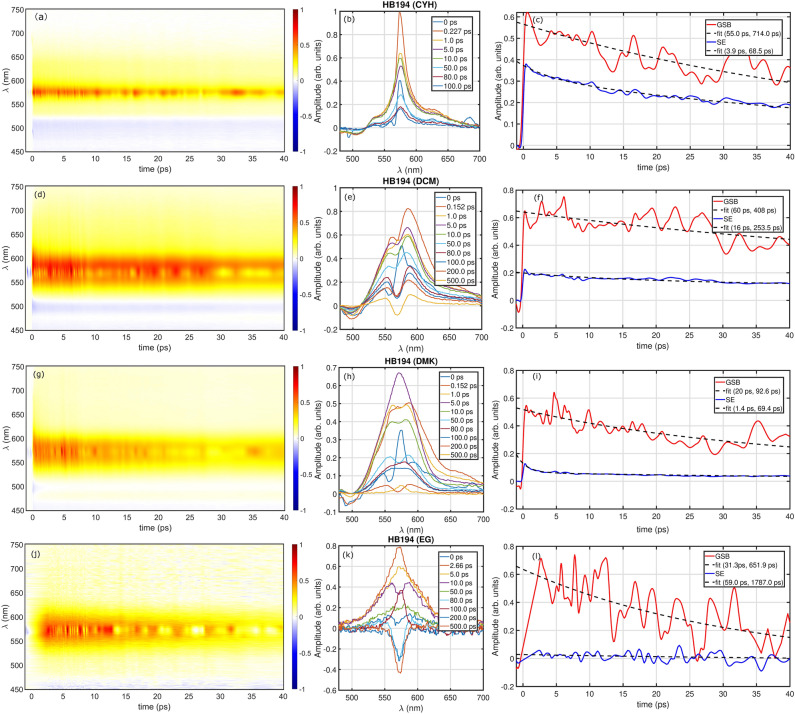
Experimental transient absorption (TA) measurements. The TA spectra of HB194 solutions in CYH (**a**), DCM (**d**), DMK (**g**) and EG (**j**) with wavelength of pump excitation at 570 nm. The transient spectra shown here are plotted as differential transmission of probe in the range of 450–750 nm. The spectral profiles at selected waiting times are plotted as solid lines in (**b**), (**e**), (**h**) and (**k**). The kinetic traces of ground state bleach (GSB) and stimulated emission (SE) are shown here as red and blue solid lines in (**c**), (**f**), (**i**) and (**l**), respectively. The instrument response function of the TA instrument was $\sim$ 120 fs.

The excited-state dynamics of HB194 is studied for 4 representative solvent molecules. In the first case, we measure the TA spectrum of HB194 (CYH), which is shown here in Fig. 2a. It shows a positive (red) band in spectra, which indicates the ground-state-bleaching (GSB) and stimulated emission (SE). The negative band (blue) represents excited-state-absorption (ESA) components. The GSB band is located at 570 nm and, based on the fluorescence spectra, the deactivation of CT state to electronically ground state is at 580 nm. The proximity of GSB and SE bands causes a strong overlap between two bands, which makes it difficult to interpret the spectroscopic results. To visualize the time-resolved spectral features, we plot the time evolution of the spectra profiles along the detection frequency axis in Fig. 2b. The time-resolved spectra are presented from initial to 100 ps. Additionally, to see the kinetic profiles, we extract the decay traces of GSB and SE bands from Fig. 2a and plot them in Fig. 2c as solid red and blue lines, respectively.

We then plot the measured TA spectrum of HB194 (DCM) in Fig. 2d. It shows a GSB band at 570 nm. As compared to the optical measurement in Fig. 2a, the significant difference is observed in the bandwidth of the prominent spectral band, which indicates a stronger interaction between the molecular system and its environment. The time dependent spectral profiles are presented in Fig. 2e from 0 to 500 ps. In this case, the fluorescence from CT to ground state is directly probed by measuring and detecting of SE at 630 nm. The traces of GSB and SE are extracted and plotted as red and blue lines in Fig. 2f. The kinetic trace clear show a remarkable difference in the lifetime (longer) compare to the ones from Fig. 2c. The TA spectrum of HB194 (DMK) is shown in Fig. 2g. The bandwidth in TA data is comparable to the results in Fig. 2d. Additionally, the time-resolved spectral profiles and extracted traces are plotted in Fig. 2h and i, respectively. The only difference is the emission band is at 650 nm, instead of 630 nm in Fig. 2f. Moreover, we show the different transient dynamics of HB194 (EG) in Fig. 2j. Firstly, it yields the relatively weak optical signal during the process of detection. The time-dependent spectral profiles are shown in Fig. 2k. The kinetic traces of GSB and SE bands at 570 and 750 nm are plotted as red and blue solid lines in Fig. 2l.

**Figure 3:**
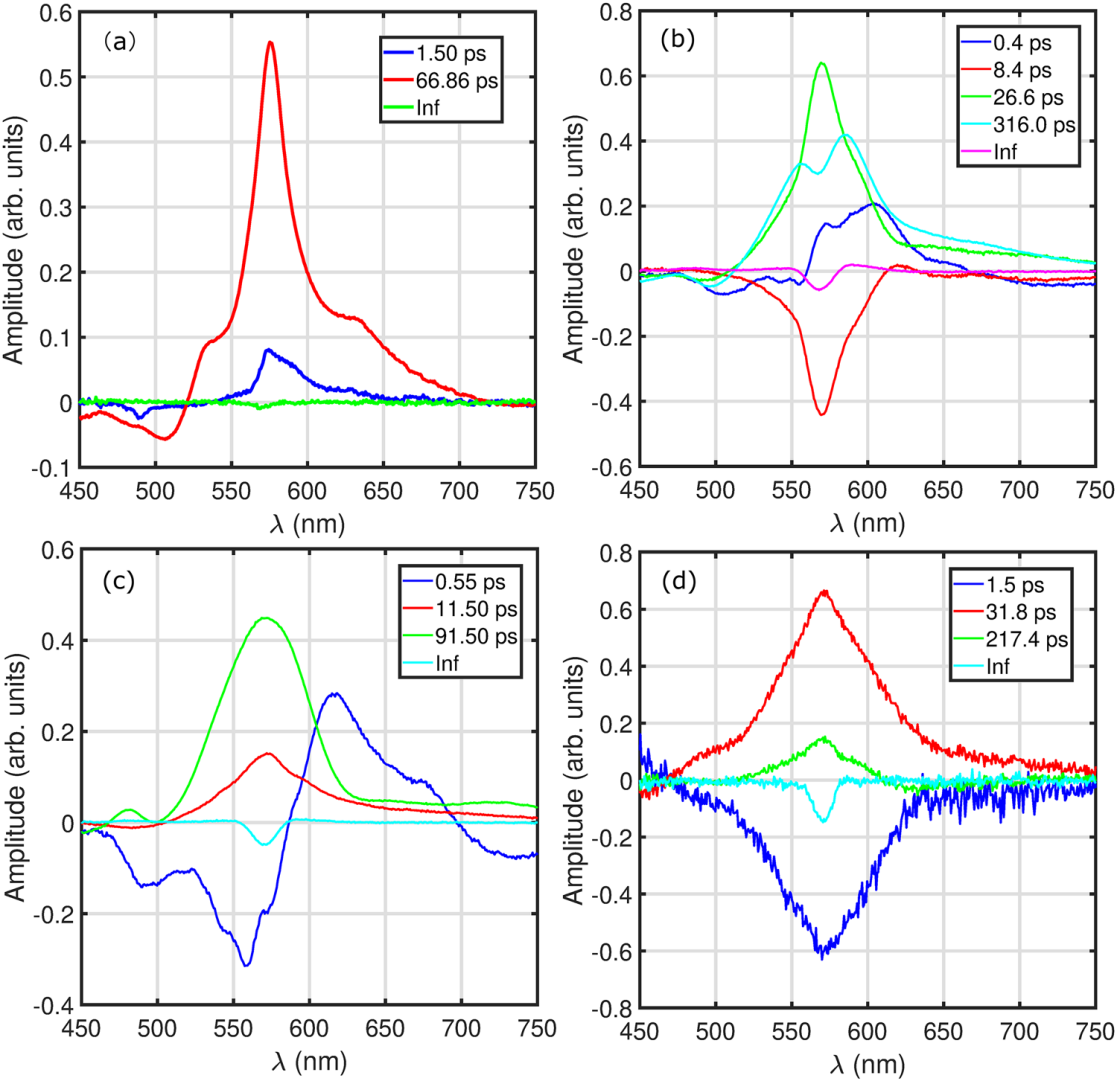
Analysis of TA data. Decay-associated spectra obtained from TA data of HB194 in different solvents: (**a**) CYH, (**b**) DCM, (**c**) DMK and (**d**) EG.

In addition to the single point kinetic-trace analysis, we employ global fitting approach to study the population dynamics along the complete detection window. For this, we firstly use the global fitting toolbox (TA analyzer version 4), which is developed by home-built Matlab code to treat the TA and two-dimensional photon echo spectra. The code analyses the TA data and yields two associated parts: time-decay traces and the decay-associated spectra (DAS). By this, we perform the data analysis of TA data and present the main results in Fig. 3 with decay lifetimes. In (a), we present the DAS of HB194 (CYH). The DAS show the spectral profiles with lifetime of 1.5 ps, 66.86 ps and infinity (“Inf”) components. The blue, red and green solid lines are plotted in (a) according to the associated decay spectra. The shortest component of 1.5 ps shows a positive peak, which locate at the central wavelength of 570 nm. It indicates a fast decay component with lifetime of 1.5 ps in TA data of Fig. 2a. The relatively slower processes of 66.86 ps in (a) (red solid line) show the dominant magnitude. In addition, the infinity part do not show a strong magnitude in DAS, which manifests the population deactivation to the electronically ground state within 67 ps.

The DAS of HB194 (DCM) are plotted in Fig. 3b. Compare to the (a), it shows 5 DAS components with relatively longer lifetimes, which can be directly confirmed from the observation of time-resolved spectral profiles in Fig. 2e. The shortest component (blue solid line) shows the lifetime of 0.4 ps. The positive magnitude at the frequency center of 600 nm manifests the fast decay of magnitude in TA data in Fig. 2d. Furthermore, the second, third and fourth components show the timescales of 8.4, 26.6 and 316.0 ps, respectively. Compare to the (a), it shows longer lifetime of excited-state dynamics in HB194 (DCM) and the deactivation from excited state to ground state are within the timescale of 316 ps. The “Inf” component show a weak peak at 570 nm (purple solid line). The global fitting results of HB194 (DMK) and (EG) are presented in Fig. 3c and d, respectively. In (c), the shortest component shows lifetime of 0.55 ps with a spectral profile of two peaks (negative and positive peaks) at 550 and 620 nm. It imply a fast increase at 550 nm and a decay at 620 nm. The second and third components show the lifetimes of 11.5 and 91.5 ps, respectively. The infinity part shows a weak peak at 570 nm. In addition, we show the DAS of HB194 (EG) in (d). The first, second and third components show the timescales of 1.5, 31.8 and 217.4 ps. A small magnitude of peak is presented in the “Inf” component in (d). On the basis of global fitting analysis, we observe the relative longer lifetime of excited-state dynamics of CT state in HB194 with solvent molecules of DCM and EG. In contrast, the HB194 (DMK) shows shorter lifetime of excited state and HB194 (CYH) shows the shortest lifetime of CT state.

### Theoretical calculations

**Figure 4:**
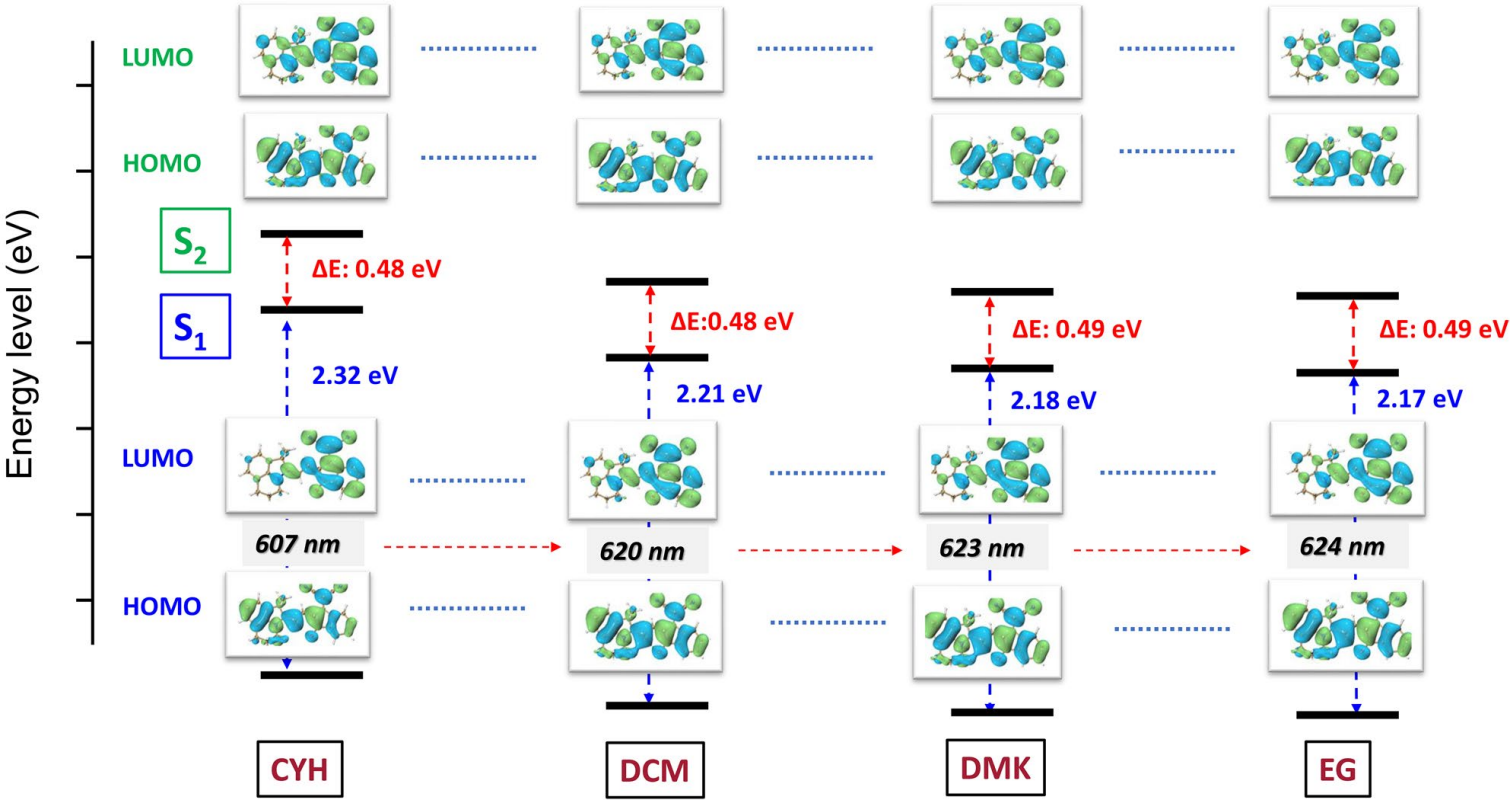
Quantum chemistry calculations. The calculated energy levels of first and second excited states, $S_{1}$ and $S_{2}$. The associated HOMO/LUMO orbitals are presented. A clear solvatochromic effect can be seen in calculated HOMO to LUMO transition.

**Figure 5:**
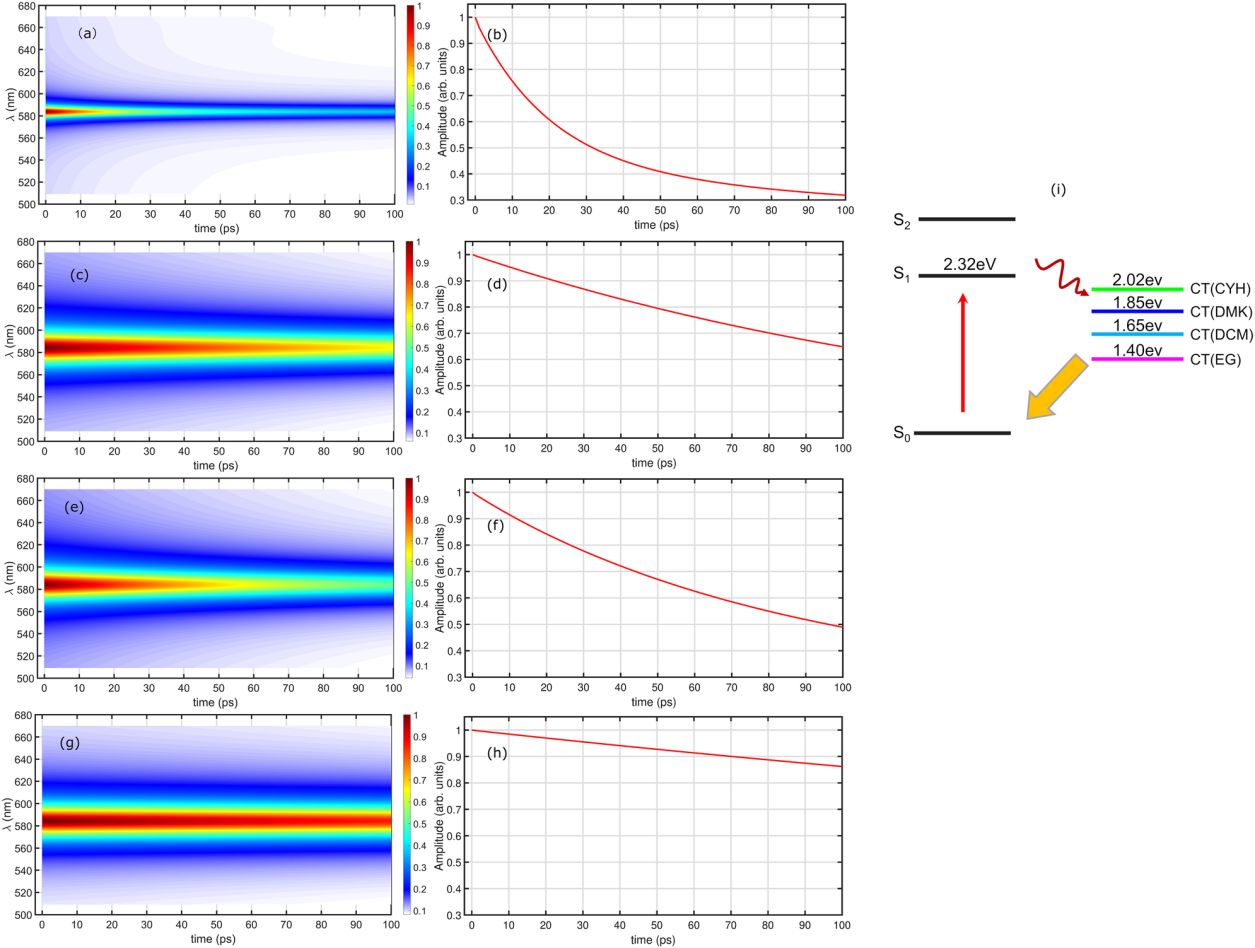
Calculation of transient absorption spectra of HB194 in different solvents. Calculated TA spectra of HB194 with CYH (**a**), DCM (**c**), DMK (**e**) and EG (**g**). The associated traces of GSB are shown in (**b**), (**d**), (**f**) and (**h**). The energy diagram employed for the calculations are presented in (**i**).

To gather further insight to the experimentally observed TA spectra and time-resolved dynamics, we perform the *ab-initio* calculations of HB194 in different solvents using linear response model. Firstly, we construct molecular structure and perform the optimization by DFT calculations (B3LYP/6-31G(d)) with the GD3BJ1 dispersion correction. The corresponding excited-state properties were studied using the time-dependent DFT (TDDFT)-PCM2-LC-BLYP*3/6-31G(d) method. Their corresponding rang-separation parameters ($\omega$, in bohr$^{-1}$) of long-range corrected LC-BLYP functional was optimally tuned through the home-made GAP-tuning procedure within the GAS-tune. All the DFT and TDDFT calculations were performed using *Gaussian* 16 code. By this, we obtain the landscape of electronically excited states and show them in Fig. 4. The two excited states of HB194 with 4 different solvents are calculated and presented. On the basis of these calculations, we plot a schematic diagram of energy levels of excited states in Fig. 5i. Moreover, the energy of CT states with different solvents is quite hard to be determine precisely. On the basis of the modeling, we calculate the transient absorption spectra of HB194 in different solvents, the calculated results are shown from Fig. 5a–h. The detailed modeling and parameters are presented in the SI.

## Discussions

The steady-state absorption and fluorescence measurements have demonstrated the significant difference of interaction strength between HB194 and solvent molecules. The ring-shape molecular structure of CYH results in a weak electric dipole (dielectric constant 2.02), which induces a weak interaction of CT state with solvent molecules. It is well known that this weak interaction do not dramatically shift energy landscape of CT state and yields a small magnitude of reorganization energy. Furthermore, on the basis of TA measurement, we observed a short lifetime of CT state in the case of HB194 (CYH) (the longest lifetime component 66 ps). However, in the case of HB194 (DCM) and (DMK), the stronger dipoles of DMK and DCM molecules induce a stronger interaction of CT state to polar environment (dielectric constants, DCM 8.93 and DMK 20.7). The relatively stronger dipole-dipole interactions result in an evident red-shift of emission peak, which yields a larger value of reorganization energy. In the schematic diagram of Fig. 5i, we plotted the energy site of CT state of HB194 (DCM) and (DMK) lower than the one of HB194 (CYH) due to the stronger electric interactions. The TA measurements and the associated global fitting results show longer lifetime of CT states. Thus, we conclude that the lower site energies of CT state reduce the speed of deactivation to the ground state.

However, the detailed mechanism of deactivation of CT states is not straightforward. It is known that the strong dipole interaction between the functional CT state and polar environment induces the structural changes of molecule, which play another important role of determining of lifetime of CT states. For this, our theoretical calculations have demonstrated that the polar environmental molecules result in small-angle distortion between donor and acceptor sides inside HB194 molecule, which slightly change the site energy of CT state. More important, this small structural changes reduce the overlap of transition dipole between CT and ground states, which is the key factor to determine the deactivation rate of CT states.

**Figure 6:**
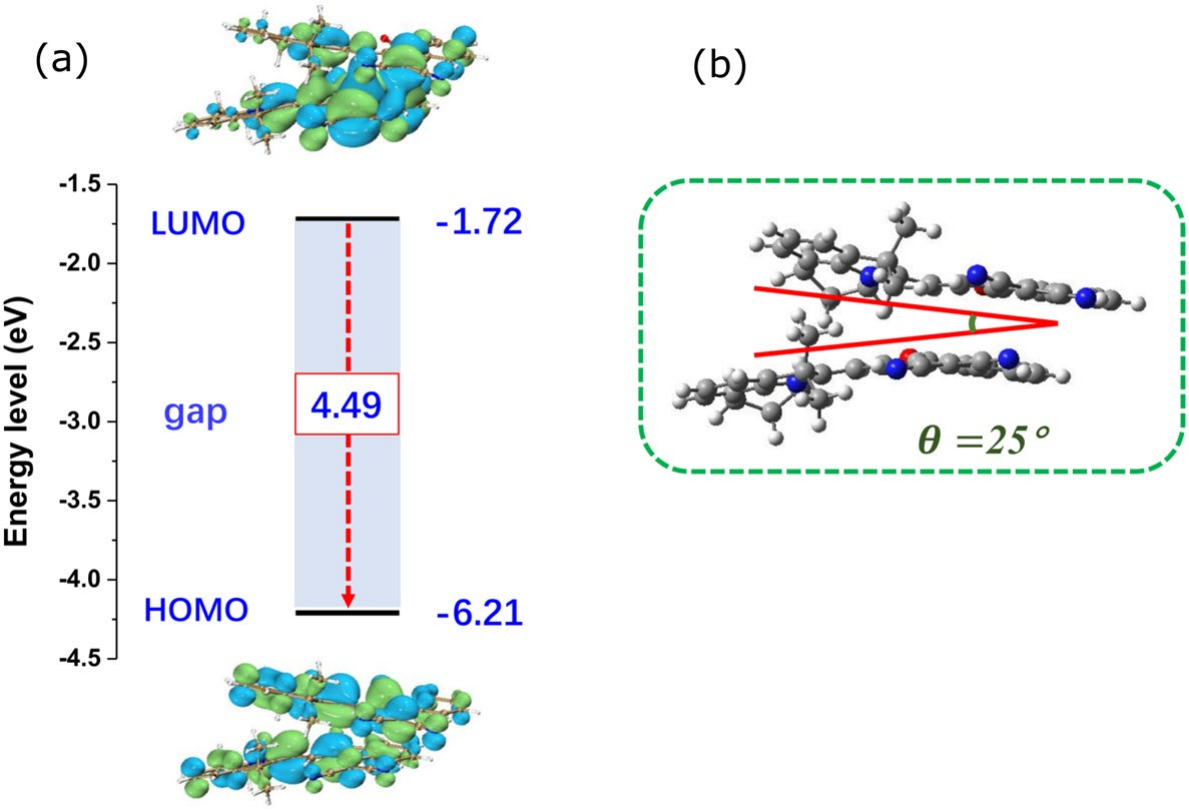
Dimer model to explain anomalous emission in EG solvent. (**a**) the excited-state energy levels with HOMO/LUMO orbitals. (**b**) molecular orientation of HB194 dimer showing the angle of 25° between the molecular planes.

The observed anomalously red-shifted emission spectrum of HB194 in ethylene glycol poses a challenge to our proposed excited state picture of the molecule. This anomaly can be explained based on known aggregation tendency of the merocyanine dyes. Numerous literature reports point to the aggregation behaviour of the merocyanine dyes^10,24,25^. Formation of dimers or higher structures of merocyanine dyes is one of the problems associated with these dyes to be used for dye-sensitized solar cells. Based on these studies, we hypothesized formation of dimers in ethylene glycol. The initial structure of two HB194 was constructed in the form of J-aggregation. The ground state of HB194 dimers are optimized and then the excited states are obtained on this basis. The optimized dimer structure constitute a inter-planar angle of 25°, as shown in Fig. 6. The dimer with this structural parameter can reproduce the experimentally observed emission frequency at 750 nm. This highlights the importance of structural parameters in the molecular aggregate of the currently studied merocyanine dye to achieve red-shifted emission. This result may be significantly useful for the community aiming to generate red emission dyes.

## Conclusions

In this paper, we studied the transient population dynamics of HB194 and its deactivation of CT states to ground state. The steady state measurements along with the quantum chemistry calculations clearly enunciate the solvatochromic behaviour of the molecule. The strong electronic interaction between CT states and polar environment induces the systematic shift of emission spectra with the solvent dielectric. To study the transient dynamics of CT states, we performed the transient absorption measurements of HB194 with different solvents. We observed that the lifetime of CT states strongly relate to the dipole-dipole interaction between system and environment. In the case of HB194 (CYH), the weak interaction to non-polar solvent results in a fast deactivation of population from CT state to the ground state. Lifetimes of the CT states are significantly prolonged to several hundreds of picosecond in the case of HB194 (DCM) and (DMK), in which the stronger interaction to the environment stabilize the energy landscape of CT states. In HB194 (EG), the possible formation of molecular dimers induces the experimentally observed anomalously large red-shifted emission peak. Our study provides an understanding towards the impact of solvent bath interactions in the photophysics of donor-bridge-acceptor based merocyanine system, specifically to the underlying CT states. We envision that the novel merocyanine dyes designed with the consideration of the role of bath interactions with CT states will pave the towards enhanced solvatochromic properties. Additionally, the bath interactions with the CT state can be used in conjunction with molecular aggregation property to design long lived red emitting dyes.

## Materials and methods

### Sample preparation

The merocyanine dye, HB194 was directly procured from Shanghai Daeyeon Chemicals Co., Ltd (homepage: http://www.shdaeyeonchem.com/) and used without further treatment. The solvents: Cyclohexane (CYH), Dichloromethane (DCM), Dimethyl Ketone (DMK), Ethylene Glycol (EG), Acetonitrile (MeCN), Tetrahydrofuran (THF) were purchased from J &K Scientific Ltd. (homepage: https://www.jkchemical.com/) To prepare the samples for optical measurements, the HB194 was directly dissolved in solvents and the solutions are diluted to optical density of 0.3 at the maximum absorption peak in steady-state absorption spectra. The UV/VIS absorption spectra were measured by UV-Vis spectrophotometer (TU1901, Beijing Purkinje General Instrument Co. Ltd.). The fluorescence spectra were detected by FluoroMax-4 spectrofluorometer (Horiba, Jobin Yvon) after subjecting the samples to excitation at 570 nm. All spectra were background corrected.

### Femtosecond transient absorption measurements

The transient absorption measurements on HB194 solutions were performed in a commercial spectrometer (Helios-EOS fire, Ultrafast System), which has been pumped by femtosecond laser (Astrella, 800 nm, 100 fs, 7 mJ/pulse, and 1 kHz repetition rate, Coherent Inc.). All experiments were carried out at room temperature and a fused silica cuvette of 1 mm path-length was used. In the spectrometer, the white light supercontinuum was generated by focusing on sapphire crystal (3 mm), which was used as a probe pulse. The pump pulse was directly generated by a commercial OPA (OPerA Solo, Coherent Inc.). The pump and probe pulses are then spatially overlapped on the sample with pump focus of 200 μm at the sample. The energy of the pump pulse for transient absorption of the sample was kept at 22.9 μJ/cm$^{2}$. The instrument response function (IRF) of this system was determined to be $\sim$ 120 fs by measuring solvent responses under the same experimental conditions. The TA data has been processed using home-written codes in MATLAB.

### Theoretical calculations

Density functional theory (DFT) calculations were performed to study the electronic structures and photo-physical properties of molecular dyes. Ground state of the molecule in different solvents were optimized at the level of B3LYP/6-31G(d) with the GD3BJ^27^ dispersion correction. The corresponding excited-state properties were studied using the time-dependent DFT (TDDFT)-PCM-LC-BLYP*/6-31G(d) method. Their corresponding rang-separation parameters ($\omega$, in bohr$^{-1}$) of long-range corrected LC-BLYP functional was optimally tuned through the home-made GAP-tuning procedure^28,29^ within the GAS-tune. All the DFT and TDDFT calculations were performed using *Gaussian* 16 code^30^. The distributions of frontier molecular orbitals were rendered using the VMD 1.9.3 program^31^. The transition dipole moment (TDM) of molecules were collected by Multiwfn program^32^.

## Supplementary Information

Supplementary Information.

## Data Availability

The data sets used and/or analyzed during the current study available from the corresponding author on reasonable request.
