# Supplementary material for: Ultrafast photoinduced dynamics of a donor-(π)bridge-acceptor based merocyanine dye
Source: Sci Rep. 2022 Oct 29;12:18216. doi: 10.1038/s41598-022-23080-5 (PMC9617881; doi:10.1038/s41598-022-23080-5)
Supplement: Supplementary file 1 — Supplementary Information. [file 41598_2022_23080_MOESM1_ESM.pdf]

Supplementary Information

Ultrafast Photoinduced Dynamics of a Donor-(π)bridge-acceptor Based Merocyanine Dye

Jianwei Shen[1], Ajay Jha[2], Meng Lv[1], Guanyu Jiang[1], Qixu Zhao[1], Zihui Liu[3], Jinquan Chen[1], Yan Yang[1], Haitao Sun[1], Hong-Guang Duan[3], Zhenrong Sun[1]
[1]*State Key Laboratory of Precision Spectroscopy, School of Physics and Electronic Science, East China Normal University, Shanghai 200241, P.R. China*
[2]*The Rosalind Franklin Institute, Rutherford Appleton Laboratory, Harwell Campus, Didcot, Oxfordshire OX11 OFA, U.K.*
[3]*Department of Physics and Institute of Modern Physics, Ningbo University, Ningbo 315211, China*

(Dated: October 28, 2022)

In this Supplementary Information, we firstly show the detailed description of global fitting approach. We then describe the treatment of extracted traces from transient absorption spectrum and the Fourier filter to polish the traces. We also show the detailed description of ab-initio calculations in this study. Theoretical calculations of transient absorption spectra and master equations will be discussed in the last section.

I. GLOBAL FITTING APPROACH AND OBTAINED RESULTS

In this section, we describe the global fitting approach, which has been used in the treatment of measured transient absorption data. The detaied description of this approach has bee shown in Ref. [1]. We firstly construct the transient absorption spectra of HB194 with different solvents. The time-resolved magnitude of each wavelength $\mathrm{A_i}(\lambda)$ has been fitted by exponential functions with the associated decay time constants τ_i. The formula employed in this calculation is given as

$$S(\lambda) = \sum_i A_i(\lambda)\mathrm{exp}(-\mathrm{T}/\tau_\mathrm{i}), \tag{S1}$$

where $\mathrm{A_i}(\lambda)$ is the decay-associated spectrum (DAS) with decay time τ_i. In the analysis, we conclude that the DAS with positive (negative) magnitude indicates the decay (increase) of amplitude in transient absorption spectrum with lifetime constant of τ_i. In this study, we performed the global fitting approach and obtained several DAS with lifetime. In principle, this global fitting can be well performed with increasing number of exponential functions. Here, we only show the best fitting quality with the minimum number of fitting functions. The obtained results are mainly presented in Fig. 3 in the main text.

II. DETAILS OF AB-INITIO CALCULATIONS

In this section, we describe the detailed calculations of HB194 with different solvents. Firstly, the molecular structure of HB194 was constructed and optimized at the level of B3LYP/6-31G(d) with the GD3BJ dispersion correction. The obtained structure of HB194 with solvents are shown in Fig. S1.

The associated excited-state properties are calculated using time-dependent DFT (TDDFT)-PCM2-LC-BLYP*3/6-31G(d) method. All the calculations have been performed using Gaussian 16 code [3]. The associated energy levels and transient dipole moment of HB194 has been calculated and shown in Fig. S2 and Fig. S3.

III. TREATMENT OF EXTRACTED TRACES

We present the detailed treatment of extracted traces in this section. We firstly select the traces at the wavelength of ground-state-bleaching and stimulated emission bands. We then reduce the noisy level by employing Fourier filter.

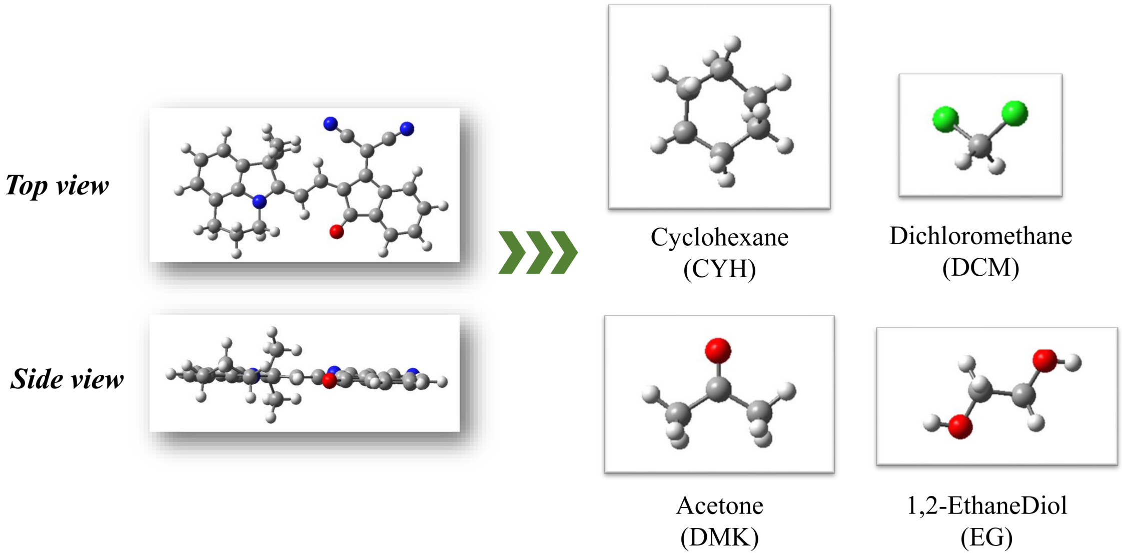

FIG. S1. The chemical structure of HB194 and the solution molecules.

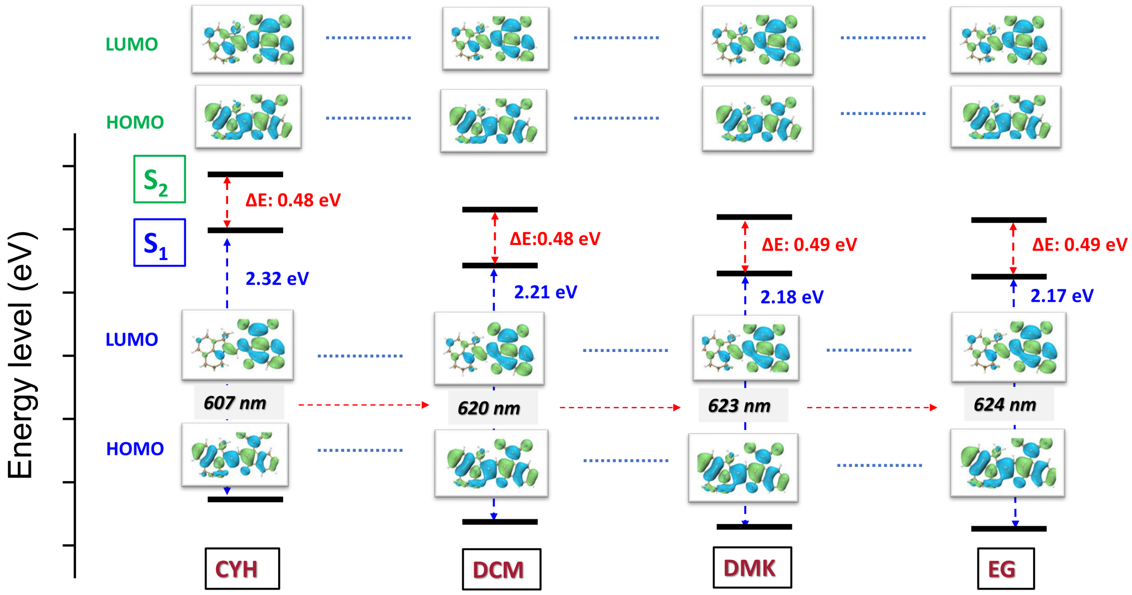

FIG. S2. Excited-state calculations with different solvents.

Here, we provide the details of Fourier transform with Tukey window. To isolate the high-frequency jitters, Fourier filtering in the frequency domain is employed to isolate each of these regions of interest with Tukey window, which shows the form

$$\omega(n) = \begin{cases} 1, & 0 \leq |n| \leq \alpha\frac{N}{2} \\ \frac{1}{2}\left(1+\cos\left[\frac{\pi(n-\alpha\frac{N}{2})}{(1-\alpha)\frac{N}{2}}\right]\right), & \alpha\frac{N}{2} \leq |n| \leq \frac{N}{2} \end{cases} \tag{S2}$$

which, due to the flat top, conserve the amplitudes of Fourier components of interest over a greater frequency range than a cosine or Gaussian window, while it still limit the artifacts arising from a pure bandpass filter. In this work, we use the Tukey window with $\alpha = 1/5$ and a Fourier bandpass filter with $\leq$700 cm^{-1}. The obtained results are shown in Fig. S4.

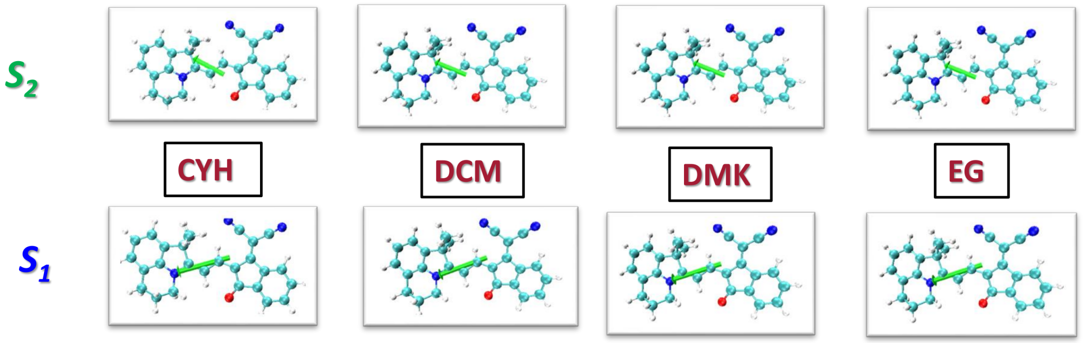

Unit(a.u.)	coordinate	CYH	DCM	DMK	EG
S_2	x	-2.39	-2.474	-2.502	-2.522
	y	1.274	1.0	0.956	0.94
	z	0.021	0.01	0.008	0.007
	total	2.708	2.668	2.678	2.691
S_1	x	-3.962	-3.91	-4.011	-4.011
	y	-1.356	-1.429	-1.329	-1.333
	z	-0.066	-0.069	-0.064	-0.064
	total	4.188	4.164	4.226	4.227

FIG. S3. The directions of transition dipole moment with different solvents (S_1 and S_2). The detailed values of transition dipole moments are listed in the table.

IV. THEORETICAL MODELING AND CALCULATIONS OF TRANSIENT ABSORPTION SPECTRUM

In this section, we show the theoretical calculations of transient absorption spectra of HB194 in different solvents. For this, we firstly construct a model Hamiltonian as following. This part will be added after decision of calculations.

V. QUANTUM MASTER EQUATION

We use time non-local method for the simulation of this paper [4, 5]. Here, we start introduction with the density matrix ρ of the total system and bath. Its time evolution is given by the Liouville-von Neumann equation with the Liouville superoperator $\mathcal{L}$ ($\hbar = 1$), such that

$$\dot{\rho} = -i[H_{\rm tot}, \rho] = \mathcal{L}\rho, \tag{S3}$$

for the total Hamiltonian $H_{\rm tot} = H_{\rm s} + H_{\rm b} + \lambda H_{\rm sb} + \lambda^2 H_{\rm ren}$, which is decomposed into the system, bath, interaction and renormalization terms. Let us consider a single degree of freedom x of the system, the bath being an ensemble of harmonic oscillators, i.e, $H_{\rm b} = \sum_{j=1}^{N} \left[p_j^2/(2m_j) + m_j\omega_j^2 x_j^2/2\right]$ and the standard form of the coupling as $H_{\rm sb} = f(x)\sum_{j=1}^{N} c_j x_j$ with some real function $f(\cdot)$.

The projection scheme of Nakajima and Zwanzig [6] allows us to separate the dynamics of the bath from that of the system. The thermal state of the bath is represented by the canonical density $\rho_{\rm b}^{\rm eq} = \exp(-\beta H_{\rm b})$ with temperature $T = (k_B\beta)^{-1}$. Applying the projector $P = \rho_{\rm b}^{\rm ER}{\rm tr}_{\rm b}$, with ${\rm tr}_{\rm b}\rho_{\rm b}^{\rm eq} = 1$ and $Q = (1 - P)$ yields the exact formal quantum

master equation for the time evolution of the reduced system density operator ρ_s [4] in the form of

$$\begin{aligned}\dot\rho_\mathrm{s}(t) &= \mathcal{L}_\mathrm{s}^\mathrm{eff}\rho_\mathrm{s}(t) + \int_0^t K(t,t')\rho_\mathrm{s}(t') + \Gamma(t),\\ \mathcal{L}_\mathrm{s}^\mathrm{eff} &= \mathcal{L}_\mathrm{s} + \lambda\,\mathrm{tr}_\mathrm{b}\mathcal{L}_\mathrm{sb}\rho_\mathrm{b}^\mathrm{eq} + \lambda^2\mathcal{L}_\mathrm{ren},\\ K(t,t') &= \lambda\,\mathrm{tr}_\mathrm{b}\mathcal{L}_\mathrm{sb}\left(\mathcal{T}e^{\int_{t'}^t Q\mathcal{L}dt''}\right)Q(\mathcal{L}_\mathrm{b}+\lambda\mathcal{L}_\mathrm{sb})\rho_\mathrm{b}^\mathrm{eq},\\ \Gamma(t) &= \lambda\,\mathrm{tr}_\mathrm{b}\mathcal{L}_\mathrm{sb}\left(\mathcal{T}e^{\int_0^t Q\mathcal{L}dt''}\right)Q\rho_\mathrm{tot}(0).\end{aligned} \tag{S4}$$

Here, $\rho_\mathrm{tot}(0)$ is the total density operator of system and bath at initial time. Moreover, we use $\mathcal{L}_\mathrm{s}$, $\mathcal{L}_\mathrm{sb}$, and $\mathcal{L}_\mathrm{ren}$ for the corresponding parts of the Liouville superoperator which are associated to the respective Hamilton operators. Moreover, $\mathcal{L}_\mathrm{s}^\mathrm{eff} = -i[H_\mathrm{s}+H_\mathrm{ren},\cdot]$ and $\mathcal{T}$ is the time-ordering operator [?]. Next, we expand the correlated thermal equilibrium state to first order in the overall coupling strength λ and obtain

$$\rho^\mathrm{eq} \approx \frac{1}{Z_\mathrm{s}}\frac{1}{Z_\mathrm{b}}e^{-\beta(H_\mathrm{s}+H_\mathrm{b})} - \lambda\frac{1}{Z_\mathrm{s}}\frac{1}{Z_\mathrm{b}}\int_0^\beta d\beta' e^{-(\beta-\beta')(H_\mathrm{s}+H_\mathrm{b})H_\mathrm{sb}^{(1)}e^{-\beta'(H_\mathrm{s}+H_\mathrm{b})}}, \tag{S5}$$

with the respective partition functions $Z_\mathrm{tot} = \mathrm{tr}\exp(-\beta H_\mathrm{tot})$, $Z_\mathrm{b} = \mathrm{tr}_\mathrm{b}\exp(-\beta H_\mathrm{b})$ and $Z_\mathrm{s} = \mathrm{tr}_\mathrm{s}\exp(-\beta H_\mathrm{s})$. Next, we take the trace over the system degrees of freedom on both sides of Eq. (S5) and get

$$\rho_\mathrm{b}^\mathrm{eq} = \frac{1}{Z_\mathrm{b}}e^{-\beta H_\mathrm{b}} + \frac{\lambda\chi}{Z_\mathrm{b}}\int_0^\beta e^{-(\beta-\beta')H_\mathrm{b}}\left(\sum_{i=1}^N c_i x_i\right)e^{-\beta' H_\mathrm{b}}. \tag{S6}$$

Here, $\chi = (1/Z_\mathrm{s})\mathrm{tr}_\mathrm{s}\left[f(x)e^{-\beta H_\mathrm{s}}\right]$. The well-known bath correlation function

$$c(t) = \int_{-\infty}^\infty \frac{d\omega}{2\pi}J(\omega)\cos(\omega t)\coth\left(\frac{\beta\omega}{2}\right) - i\int_{-\infty}^\infty\frac{d\omega}{2\pi}J(\omega)\sin(\omega t) \equiv a(t) - ib(t) \tag{S7}$$

is given in terms of the standard bath spectral density $J(\omega)$ and has the real part $a(t)$ and imaginary part $b(t)$. Inserting Eqs. (S5) and (S6) into Eq. (S4), we can express the last three terms of Eq. (S4) by $a(t)$ and $b(t)$ according to

$$\begin{aligned}\mathcal{L}_\mathrm{s}^\mathrm{eff} &= \mathcal{L}_\mathrm{s} + \lambda^2\mu\mathcal{L}_\mathrm{ren,s} + \lambda^2\chi\mu\mathcal{L}^-,\\ K(t,t') &= \lambda^2\mathcal{L}^-\left(a(t-t')\mathcal{T}e^{\int_{t'}^t\mathcal{L}_\mathrm{s}}\mathcal{L}^- + b(t-t')\mathcal{T}e^{\int_{t'}^t\mathcal{L}_\mathrm{s}}\mathcal{L}^+\right),\\ \Gamma(t) &= \lambda^2\mathcal{L}^-\int_{-\infty}^0 dt'\left[a(t-t')\mathcal{T}e^{\int_{t'}^t\mathcal{L}_\mathrm{s}}\mathcal{L}^-\rho_\mathrm{s}^\mathrm{eq} + b(t-t')\mathcal{T}e^{\int_{t'}^t\mathcal{L}_\mathrm{s}}\mathcal{L}^+\rho_\mathrm{s}^\mathrm{eq}\right],\end{aligned} \tag{S8}$$

with $\mathcal{L}^- = -i[H_\mathrm{sb},\cdot]$ and $\mathcal{L}^+ = [H_\mathrm{sb},\cdot]_+ - 2\chi$. In terms of the spectral density, the potential renormalization is given by $\mu = \int_{-\infty}^\infty \frac{d\omega}{2\pi}J(\omega)/\omega$.

In order to obtain an analytic form of the bath correlation function, any given spectral density (in our particular case, we use the standard Ohmic form) can be approximated by a sum of Lorentzian-like spectral terms [? ?] according to

$$J(\omega) = \frac{\pi}{2}\sum_{k=1}^n \frac{p_k\omega}{[(\omega+\Omega_k)^2+\Gamma_k^2][(\omega-\Omega_k)^2+\Gamma_k^2]}, \tag{S9}$$

where the spectral amplitude p_k, the frequency Ω_k and the width Γ_k following from the expansion of the original function in terms of the Lorentzian-like terms. Using the expanded form in Eq. (S7), we get

$$\begin{aligned}a(t) &= \sum_{k=1}^n\frac{p_k}{8\Omega_k\Gamma_k}\coth\left[\frac{\beta}{2}(\Omega_k+i\Gamma_k)e^{i\Omega_k t-\Gamma_k t}\right] + \sum_{k=1}^n\frac{p_k}{8\Omega_k\Gamma_k}\coth\left[\frac{\beta}{2}(\Omega_k-i\Gamma_k)e^{-i\Omega_k t-\Gamma_k t}\right] + \frac{2i}{\beta}\sum_{k=1}^{n'}J(i\nu_k)e^{-\nu_k t},\\ b(t) &= \sum_{k=1}^n\frac{ip_k}{8\Omega_k\Gamma_k}\left(e^{i\Omega_k t-\Gamma_k t} - e^{-i\Omega_k t-\Gamma_k t}\right),\end{aligned} \tag{S10}$$

with the Matsubara frequencies $\nu_k = 2\pi k/\beta$.

Next, we rewrite the correlation functions as $a(t) = \sum_{k=1}^{n_r} \alpha_k^r e^{\gamma_k^r t}$ and $b(t) = \sum_{k=1}^{n_i} \alpha_k^i e^{\gamma_k^i t}$ with $n_i = 2n$, $n_r = 2n+n'$, where n' is the number of Matsubara frequencies used. Then, we define new auxiliary "density matrices" which incorporate both memory effects and initial correlations according to

$$\begin{aligned}
\rho_k^r(t) &= \lambda \left(\mathcal{T} e^{\int_0^t dt' \mathcal{L}_s} e^{\gamma_k^r t} \int_0^\infty dt' e^{\mathcal{L}_s t'} e^{\gamma_k^r t'} \mathcal{L}^- \rho_s^{eq} + \int_0^t dt' e^{\gamma_k^r (t-t')} \mathcal{T} e^{\int_{t'}^t L_s} \mathcal{L}^- \rho_s(t') \right), \\
\rho_k^i(t) &= \lambda \left(\mathcal{T} e^{\int_0^t dt' \mathcal{L}_s} e^{\gamma_k^i t} \int_0^\infty dt' e^{\mathcal{L}_s t'} e^{\gamma_k^i t'} \mathcal{L}^+ \rho_s^{eq} + \int_0^t e^{\gamma_k^i (t-t')} \mathcal{T} e^{\int_{t'}^t dt' L_s} \mathcal{L}^+ \rho_s(t') \right).
\end{aligned} \tag{S11}$$

The time-retarded Eq. (S4) (first term) can then be deconvoluted into the coupled first-order equations

$$\begin{aligned}
\dot{\rho}_s(t) &= \mathcal{L}_s^{eff}(t)\rho_s(t) + \lambda \left[\sum_{k=1}^{n_r} \alpha_k^r \mathcal{L}^- \rho_k^r(t) + \sum_{k=1}^{n_i} \alpha_k^i \mathcal{L}^- \rho_k^i(t) \right], \\
\dot{\rho}_k^r(t) &= (\mathcal{L}_s(t) + \gamma_k^r)\rho_k^r(t) + \lambda \mathcal{L}^- \rho_s(t), k = 1, \ldots, n_r, \\
\dot{\rho}_k^i(t) &= (\mathcal{L}_s(t) + \gamma_k^i)\rho_k^i(t) + \lambda \mathcal{L}^+ \rho_s(t), k = 1, \ldots, n_i.
\end{aligned} \tag{S12}$$

This is the time non-local quantum master equation which we use to calculate the dynamics of the quantum system.

---

[1] Prokhorenko, V. I. *European Photochemistry Association Newletter* June 2012, p21.
[2] Peterman, E. J. *et al.* The nature of the excited state of the reaction center of photosystem II of green plants: A high-resolution fluorescence spectroscopy study. Proc. Natl. Acad. Sci. (USA) **95**, 6128-6133 (1998).
[3] M. J. Frisch, G. W. Trucks, H. B. Schlegel, G. E. Scuseria, M. A. Robb, J. R. Cheeseman, G. Scalmani, V. Barone, G. A. Petersson, H. Nakatsuji, X. Li, M. Caricato, A. Marenich, J. Bloino, B. G. Janesko, R. Gomperts, B. Mennucci, H. P. Hratchian, J. V. Ortiz, A. F. Izmaylov, J. L. Sonnenberg, D. Williams-Young, F. Ding, F. Lipparini, F. Egidi, J. Goings, B. Peng, A. Petrone, T. Henderson, D. Ranasinghe, V. G. Zakrzewski, J. Gao, N. Rega, G. Zheng, W. Liang, M. Hada, M. Ehara, K. Toyota, R. Fukuda, J. Hasegawa, M. Ishida, T. Nakajima, Y. Honda, O. Kitao, H. Nakai, T. Vreven, K. Throssell, J. A. Montgomery, Jr., J. E. Peralta, F. Ogliaro, M. Bearpark, J. J. Heyd, E. Brothers, K. N. Kudin, V. N. Staroverov, T. Keith, R. Kobayashi, J. Normand, K. Raghavachari, A. Rendell, J. C. Burant, S. S. Iyengar, J. Tomasi, M. Cossi, J. M. Millam, M. Klene, C. Adamo, R. Cammi, J. W. Ochterski, R. L. Martin, K. Morokuma, O. Farkas, J. B. Foresman, D. J. Fox, Gaussian 16: Revision A.03 (Gaussian Inc., 2016).
[4] C. Meier, and D. J. Tannor, J. Chem. Phys. **111**, 3365 (1999).
[5] U. Kleinekathöfer, J. Chem. Phys. **121**, 2505 (2004).
[6] R. Zwanzig, *Lectures in Theoretical Physics*, Boulder, Colorado (Interscience, New York, 1961), Vol. 3.

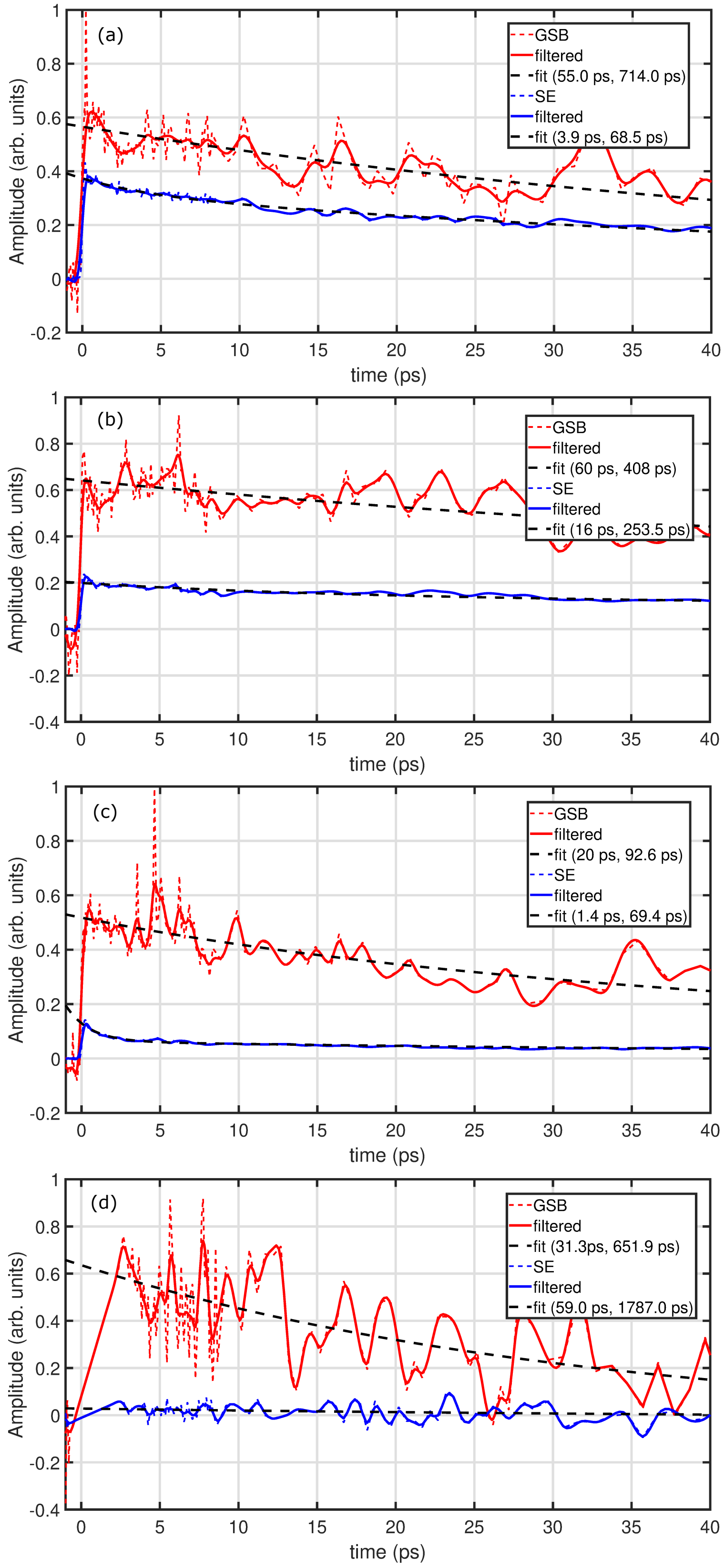

FIG. S4. The traces of GSB and SE from CYH (a), DCM (b), DMK (c) and EG (d). The raw data of traces are plotted as red dashed lines and the filtered traces are shown as red solid lines. The curves are fitted by two exponential functions and the decay timescales are presented.
